# The reasons for ceramic-on-ceramic revisions between the third- and fourth-generation bearings in total hip arthroplasty from multicentric registry data

**DOI:** 10.1038/s41598-021-85193-7

**Published:** 2021-03-10

**Authors:** Sang-Min Kim, Kee Hyung Rhyu, Jeong Joon Yoo, Seung-Jae Lim, Je Hyun Yoo, Suc Hyun Kweon, Kyung-Jae Lee, Seung-Beom Han

**Affiliations:** 1 Department of Orthopedic Surgery, Korea University College of Medicine, Guro Hospital, Seoul, South Korea; 2 Department of Orthopedic Surgery, Kyung Hee University Hospital, College of Medicine, Kyung-Hee University, Seoul, South Korea; 3 Department of Orthopedic Surgery, Seoul National University College of Medicine, Seoul, South Korea; 4 Department of Orthopedic Surgery, Samsung Medical Center, Sungkyunkwan University School of Medicine, Seoul, South Korea; 5 Department of Orthopedic Surgery, Hallym University Sacred Heart Hospital, Anyang, South Korea; 6 Department of Orthopedic Surgery, Wonkwang University School of Medicine, Iksan, South Korea; 7 Department of Orthopedic Surgery, Dongsan Medical Center, Kyemyung University School of Medicine, Daegu, South Korea; 8 Department of Orthopedic Surgery, Korea University College of Medicine, Anam Hospital, 73 Inchon-ro, Seongbuk-gu, Seoul, 02841 South Korea

**Keywords:** Health care, Materials science

## Abstract

This study aimed to evaluate (1) the overall reasons for first revision in CoC THAs; (2) whether the reasons for revision differ between third-generation and fourth-generation CoC THAs; and (3) the specific factors associated with bearing-related problems as the reason for revision. We retrospectively reviewed 2045 patients (2194 hips) who underwent first revision THA between 2004 and 2013, among which 146 hips with CoC bearings underwent revision. There were 92 hips with third-generation ceramic bearings and 54 hips with fourth-generation ceramic bearings. The major reasons for CoC THA revisions were ceramic fracture and loosening of the cup or stem. When ceramic fracture, squeaking, incorrect ceramic insertion, and unexplained pain were defined as directly related or potentially related to ceramic use, 28.8% (42/146) of CoC revisions were associated with bearing-related problems. Among the third-generation ceramic bearings, revision was performed in 41.3% (38/92) of cases owing to bearing-related problems whereas revisions were performed for only 7.4% (4/54) of cases with fourth-generation ceramic bearings owing to bearing-related problems (*p* < 0.001). Younger age, lower American Society of Anesthesiologists (ASA) grade, and preoperative diagnosis of osteonecrosis were factors related to CoC THA revisions due to bearing-related problems.

## Introduction

Ceramic-on-ceramic (CoC) bearing was introduced in total hip arthroplasty (THA) in the early 1970s because its properties of high-wear resistance and biocompatibility were superior to those of the alloys and polymers in use at the time^1^. However, the early prostheses had high failure rates as a result of poor acetabular fixation and component fracture related to design flaws and material processing^2,3^. Important advances in the material properties including the purity, density, and grain structure have increased the durability of alumina ceramics (Biolox Forte; CeramTec AG, Plochingen, Germany). Although the third-generation of alumina ceramics has showed promising results in CoC THA, concerns such as limited sizing options, stripe wear, breakage and squeaking persist^4–6^.

Further improvements in the mechanical and wear performance led to the development of the fourth-generation of CoC bearing (Biolox Delta; CeramTec AG). Owing to increased toughness and burst strength, it was expected that fractures would occur with much less frequency than the reported fracture rate of the third-generation ceramics. In addition, Biolox Delta heads are offered in a wider range of sizes, which provides greater options for offset and leg length control.

Despite the increased use of CoC bearings in recent years, the reasons behind the need for revisions of CoC THAs are not well understood. Among 38 CoC THA revisions investigated by Massin et al.^7^, femoral loosening (13 of 38) was the main reason for reoperation. A 10-year minimum follow-up data of 301 CoC THAs showed that out of nine revisions, four occurred due to periprosthetic femoral fracture^8^. In general, there is also limited information regarding CoC revisions in the national registry data^9–11^. Among 11,096 patients from the Danish hip arthroplasty registry, only 71 CoC THAs were identified^12^.

We therefore sought to determine (1) the overall reasons for first revision in CoC THAs; (2) whether the reasons for revision differ between the third- and fourth-generation CoC THAs; and (3) the specific factors related to bearing problems as the reason for revision.

## Materials and methods

Data from our local total hip replacement registry, to which eight orthopaedic surgeons report was utilized after obtaining approval from the Institutional Review Board (IRB) (KUH1060140). The informed consent was waived by the IRB (KUH1060140). All methods was carried out in accordance with relevant guidelines and regulations. Each institution’s database containing prospectively collected demographics, surgical data, and patient outcomes identified 2045 patients (2194 hips) who underwent first revision THA during the same interval (2004–2013), in which 169 hips (6.7%) with CoC bearings were revised.

Ten patients (10 hips) were lost to follow-up, and two patients (2 hips) died before the minimum 5-year postoperative follow-up. Eight patients (8 hips) undergoing CoC THA with a sandwich liner were excluded from this study. No hips had a forte head with a delta insert or a delta head with a forte insert. Three patients (3 hips) were dropped as a result of insufficient data. Thus, 146 patients (146 hips) were included in the final cohort. During the same period, 13,023 primary THA with CoC bearings had been performed.

There were 92 hips (63.0%) with the third-generation (Biolox Forte) bearing and 54 hips (37.0%) with the fourth-generation (Biolox Delta) bearing. The diameter of the CoC bearing was 28 mm in 67 hips (45.9%), 32 mm in 42 hips (28.8%), and 36 mm in 37 hips (25.3%). Mean age was 58.0 ± 23.3 years (range 23–91 years), with a male proportion of 54.8% (80/146). The mean follow-up period was 8.3 ± 5.9 years (range 5.0–16.8 years).

Detailed demographic data for each patient, including age, sex, body mass index, preoperative diagnosis, comorbidities, and physical status^13,14^, were obtained. Characteristics of the surgery, such as operation time, method of anesthesia, surgical approach, type of surgery, and interval between initial surgery and revision were also investigated.

Reasons for revision were classified into three groups—directly related to ceramic use, potentially related to ceramic use, and not specific to ceramic use^15^. Reasons directly related to ceramic use included impingement, ceramic fracture, squeaking, and incorrect ceramic insert insertion. Those potentially related to ceramic use included iliopsoas irritation and unexplained pain. Those not specific to ceramic use included loosening, infection, instability, periprosthetic fracture, osteolysis, breaking of non-ceramic components, tumor, and surgical technical errors other than those related to the insertion of the ceramic. Reasons directly or potentially related to ceramic use were defined as bearing-related problems, whereas those not specific to ceramic use were defined as bearing-independent problems.

### Statistical analysis

Basic descriptive statistical analyses were used to describe the study population. Values were expressed as means or percentages. The independent *t*-test was used to compare continuous variables, and the Mann–Whitney test or Fisher’s exact test was used to compare categorical variables. Statistical significance was defined at *p* ≤ 0.05. Statistical Package for the Social Sciences software ver.18.0 (SPSS Inc., Chicago, IL, USA) was used for all statistical analyses.

## Results

Patient demographics are shown in Table 1. Baseline demographics were similar between the third- and fourth-generation CoC revision groups. Of the surgical characteristics, the type of surgery and interval between initial surgery and revision were significantly different between the two groups (Table 2). In the third-generation CoC revision group, bearing change was more frequently performed [27/92, 29.3% vs*.* 5/54, 9.2%, *p* = 0.045] and the time to revision was longer (58.9 ± 47.6 vs. 27.5 ± 12.4 months, *p* < 0.001), compared to the fourth-generation CoC revision group.

**Table 1:** Baseline patients characteristics.

	Biolox forte (n = 92)	Biolox delta (n = 54)	*p*
Age (years)	59.0 ± 13.7	62.0 ± 15.8	0.113
Female sex	40 (43.5%)	26 (48.1%)	0.606
Body mass index (kg/m^2^)	24.8 ± 3.5	24.0 ± 3.6	0.205
**ASA classification**			0.970
I or II	78 (84.8%)	46 (85.2%)	
III or IV	14 (15.2%)	8 (14.8%)	
**Charlson comorbidity index**			0.832
1 or 2	65 (70.7%)	38 (70.4%)	
3 or 4	24 (26.1%)	14 (25.9%)	
≥ 5	3 (3.3%)	2 (4.7%)	
**Preoperative diagnosis**			0.440
Osteonecrosis	50 (54.3%)	18 (33.3%)	
Osteoarthritis	13 (14.1%)	14 (25.9%)	
Fracture of the femoral neck	11 (12.0%)	13 (24.1%)	
Others	18 (19.6%)	9 (16.7%)	
Follow-up period (mos)	83.3 ± 17.7	76 ± 14.2	0.080

**Table 2:** Characteristics of surgery.

	Biolox forte (n = 92)	Biolox delta (n = 54)	*p*
Operation time (min)	189.7 ± 43.3	199.3 ± 38.4	0.412
**Anesthesia method**			0.709
General	68 (73.9%)	38 (70.4%)	
Spinal	24 (26.1%)	16 (29.6%)	
**Surgical approach**			0.544
Anterolateral	18 (19.6%)	8 (14.8%)	
Direct lateral	14 (15.2%)	8 (14.8%)	
Posterolateral	60 (65.2%)	38 (70.4%)	
**Type of surgery**			0.045
Bearing change, only	27 (29.3%)	5 (9.2%)	
Partial revision (Cup or Stem)	51 (55.5%)	40 (74.1%)	
Total revision	14 (15.2%)	9 (16.7%)	
Interval between initial surgery and revision (mos)	58.9 ± 47.6	27.5 ± 12.4	< 0.001

The reasons for CoC revision in decreasing order were ceramic fracture in 34 hips, loosening of cup or stem in 34 hips, periprosthetic fracture in 24 hips, infection in 21 hips, instability in 19 hips, noise in 6 hips, malposition in 3 hips, leg length discrepancy in 1 hip, osteolysis in 1 hip, tumor in 1 hip, and unexplained pain in 1 hip.

**Figure 1:**
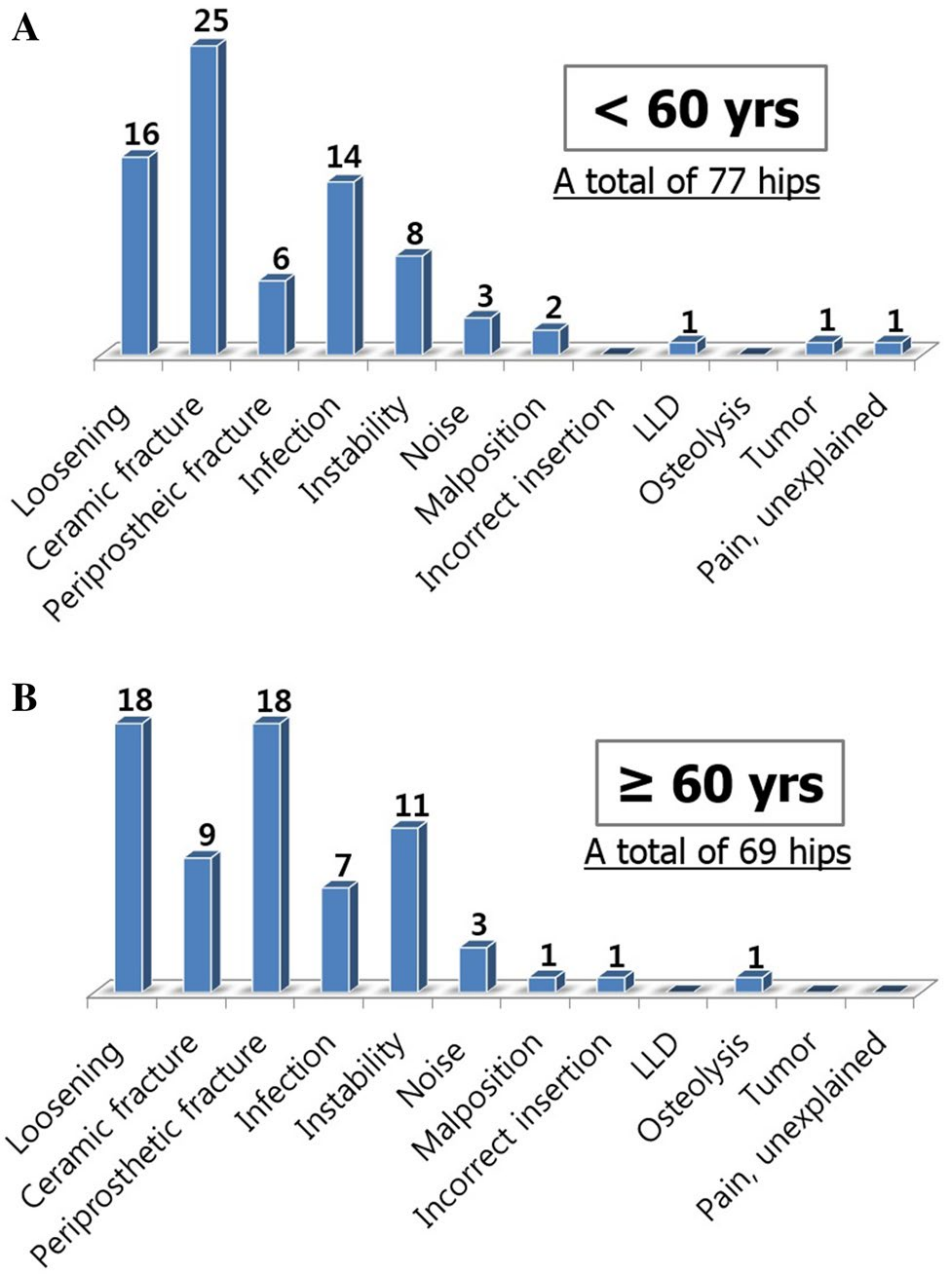
(**A**,**B**) The reasons for CoC THA revisions according to age.

**Figure 2:**
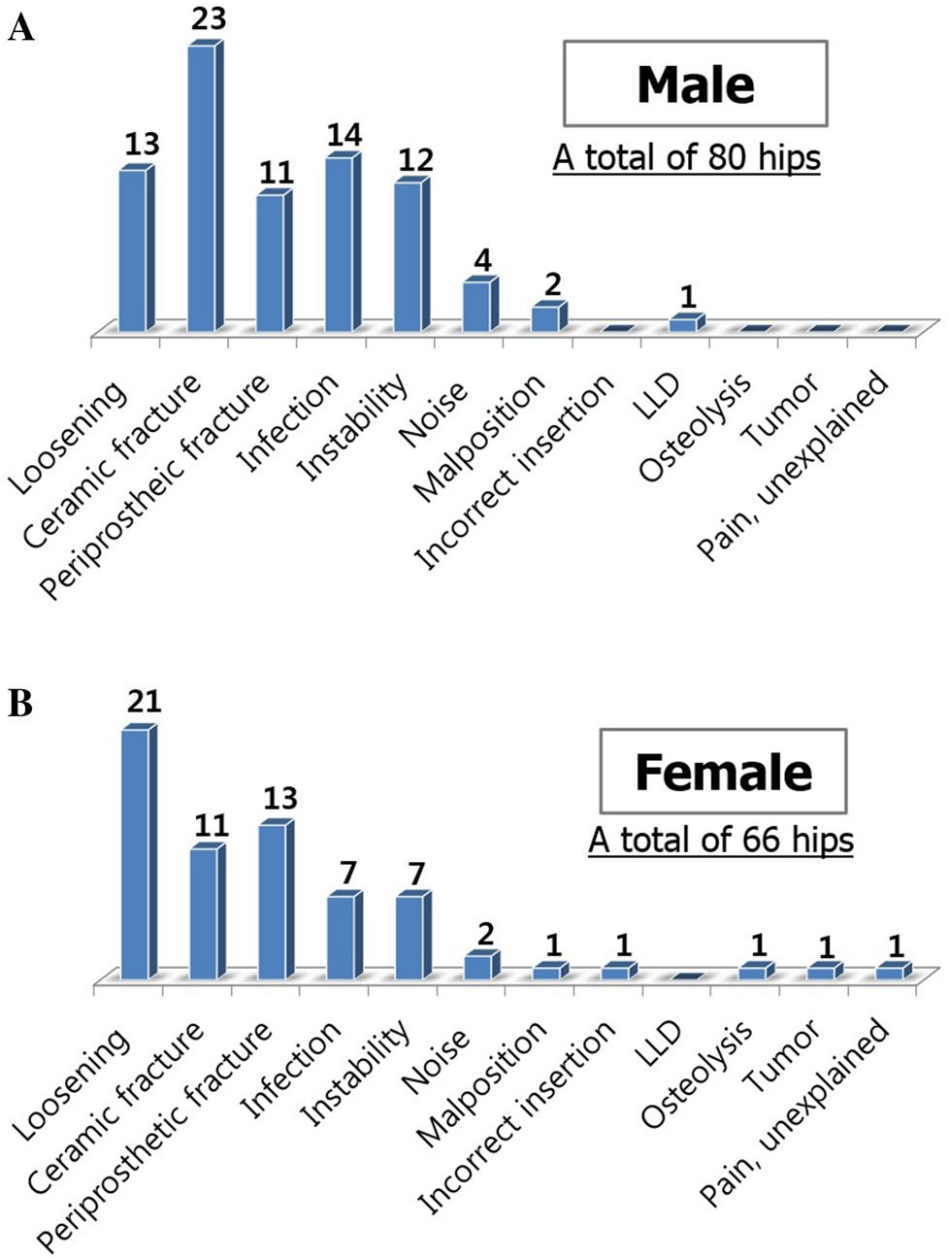
(**A**,**B**) The reasons for CoC THA revisions according to gender.

**Figure 3:**
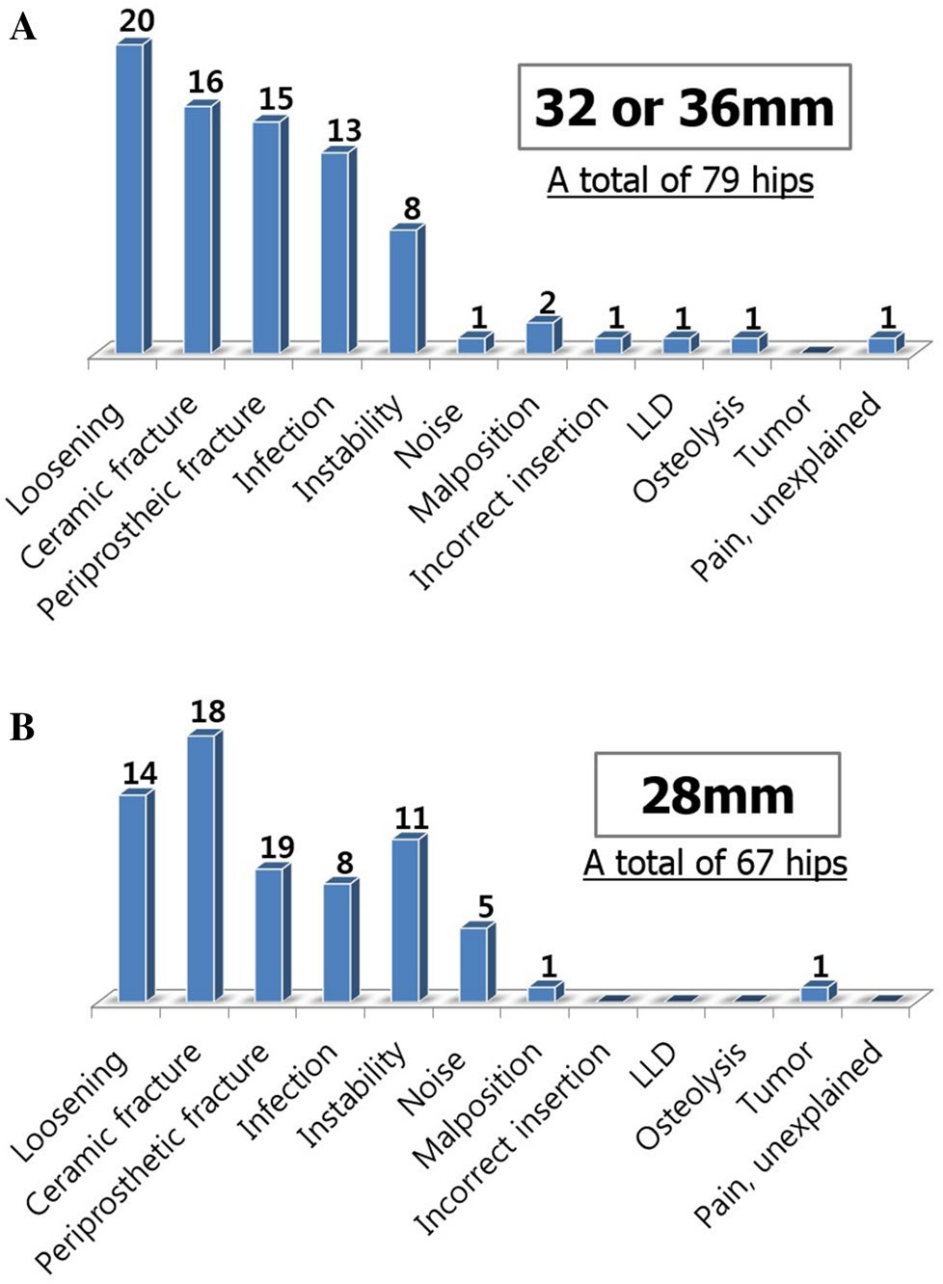
(**A**,**B**) The reasons for CoC THA revisions according to the femoral head diameter.

The reasons for revision were analyzed in greater detail, with subgroups according to age, gender, and ceramic diameter. In the patients aged < 60 years, ceramic fracture was a major reason for CoC revision whereas loosening and periprosthetic fracture were major reasons in patients aged ≥ 60 years (Fig. 1). Gender differences were also noted. Male patients underwent revisions mainly due to ceramic fracture, while female patients received revision mainly due to loosening (Fig. 2). According to the diameter of the ceramic bearing, ceramic fracture was a major reason for 28 mm-head THA revisions, whereas prosthesis loosening was a major reason for 32 or 36 mm-head THA revisions (Fig. 3).

Forty-two (28.8%) CoC revisions were associated with bearing-related problems, with a significant difference in their proportion among the reasons for revision between the third-generation and fourth-generation CoC THA groups [38/92 (41.3%) vs. 4/54 (7.4%), *p* < 0.001] (Table 3). Ceramic fracture was the most common cause of bearing-related problems [34/42 (81.0%)]. Fractures of the femoral head were more common than those of the acetabular insert [20/34(58.8%) vs. 14/34(41.2%)], though all four cases among the fourth-generation CoC THAs were fractures of the acetabular insert.

**Table 3:** Comparison on reasons for CoC revisions between third and fourth generation.

	Biolox forte (n = 92)	Biolox delta (n = 54)
**Bearing-related problems**		
Ceramic fracture	31	3
Noise	6	0
Incorrect ceramic insertion	0	1
Pain, unexplained	1	0
Total	38 (41.3%)	4 (7.4%)
**Bearing-independent problems**		
Loosening of cup or stem	24	10
Periprosthetic fracture	10	14
Infection	6	15
Recurrent dislocation	10	9
Leg length discrepancy	1	0
Malposition	1	2
Osteolysis	1	0
Tumor	1	0
Total	54 (58.7%)	50 (92.6%)

The CoC THA revisions due to bearing-related problems were performed at an average of 50.2 months (range 1–159 months). Of 42 bearing-related revisions, 10 (23.8%) occurred within 2 years and 17 (40.5%) occurred within 5 years. Younger age, lower American Society of Anesthesiologists (ASA) grade, and preoperative diagnosis of osteonecrosis were significantly associated with CoC revisions due to bearing-related problems (Table 4).

**Table 4:** Factors associated with bearing-related problems.

	Bearing related problems (n = 42)	Bearing-independent problems (n = 104)	*p*
**Age**			0.010
< 60 years	29 (69.0%)	48 (46.2%)	
≥ 60 years	13 (31.0%)	56 (53.8%)	
**Sex**			0.052
Male	27 (64.3%)	53 (51.0%)	
Female	15 (35.7%)	51 (49.0%)	
**Affected side**			0.141
Right	25 (59.5%)	53 (51.0%)	
Left	17 (40.5%)	51 (49.0%)	
**ASA grade**			0.034
I or II	37 (88.1%)	77 (74.0%)	
III or IV	5 (11.9%)	27 (26.0%)	
**Charlson comorbidity index**			0.066
1 or 2	34 (81.0%)	80 (76.9%)	
≥ 3	8 (19.0%)	24 (23.1%)	
**Preoperative diagnosis**			< 0.001
Osteonecrosis	29 (69.0%)	39 (38.0%)	
Others	13 (31.0%)	65 (62.0%)	
**Anesthesia method**			0.841
General	27 (64.3%)	69 (66.3%)	
Regional	15 (35.7%)	35 (33.7%)	
**Surgical approach**			0.816
Anterolateral	8 (19.0%)	18 (17.3%)	
Direct lateral	10 (23.8%)	12 (11.5%)	
Posterolateral	24 (57.2%)	74 (71.2%)	
**Ceramic diameter**			0.621
32 or 36 (mm)	29 (69.0%)	50 (48.1%)	
28 (mm)	13 (31.0%)	54 (51.9%)	

## Discussion

The reasons for revision of metal-on-polyethylene (MoP) THAs have typically related to wear and osteolysis and metal-on-metal (MoM) revision surgery has been performed mainly for adverse reaction to metal debris. In contrast, it remains uncertain why contemporary CoC THAs lead to failure. Moreover, it has not been fully clear whether the two types of currently used CoC bearings are associated with different reasons for revision. In the current study, the most common reason for first revision in CoC THA was ceramic fracture (34/146, 23.3%). When the reasons directly or potentially related to ceramic use were defined as bearing-related problems, the third-generation CoC THA revisions were associated with a higher proportion of bearing-related problems than the fourth-generation CoC THAs [38/92 (41.3%) *vs.* 4/54 (7.4%), *p* < 0.001].

While this study included a large series of CoC THA revisions, it has some limitations. First, this study was performed at nine tertiary centers, so there may have been some differences in the surgical procedures including skin incision, surgical approach, or implantation technique. Although all surgeons were experts with more than 5 years of experience in adult reconstruction, a potential bias might have been present. However, the large cohort size of this study may render the results more significant. One hundred and forty-six of CoC revisions are not a small number, considering even the national registry data include less than 100 CoC THA revisions^12^. Second, various types of prostheses were used, which limits comparisons between different prostheses. The contribution of the characteristics of prostheses to revision was neglected in this study. In addition, a majority of 28 mm femoral head were included in the third generation than in the fourth due to surgical skill evolution. This could be an important bias to the study. Nevertheless, our results provide valuable data that cannot be gleaned from the national registry. Specifically, reasons for revision were classified into three subgroups: directly related to ceramic use, potentially related to ceramic use, and not specific to ceramic use and all causes of failure were considered. Third, the retrospective nature of this study has an inherent risk of observer bias, including the potential for missing data and the inability to control confounding variables. Fourth, the outcome of CoC revisions was not evaluated. There are no data regarding radiographic parameters such as inclination and anteversion angles and clinical scores. This study did not aim to assess the outcome of CoC THA revisions, but sought to investigate the reasons for revision surgery. Lastly, multivariable analyses were not performed to identify factors associated with CoC revisions due to bearing-related problems because the sample size was not sufficient to consider all variables.

In the third-generation CoC revision group, bearing change was more frequently performed, and it occurred mainly due to ceramic fracture. Overall, the third-generation CoC bearing was more associated with bearing-related problems than the fourth-generation CoC bearing. In the case of ceramic fracture, a change in the bearing type to MoP is not recommended due to concerns over metallosis^16^. The fourth-generation CoC revision group showed a shorter time to revision compared to the third-generation CoC revision group. Based on our results, bearing-independent problems such as loosening, infection, dislocation, and periprosthetic fracture were the dominant reasons for revision of the fourth-generation CoC THAs with these issues often occurring within 2 years postoperatively.

The ceramic fracture remains an ongoing issue in CoC THAs, although the fracture rates of ceramics have decreased over time with improvements in manufacturing processes and materials. In the early-generation ceramics, it was possible for crack propagation to result in fracture, but the incorporation of zirconia into the alumina matrix (Biolox Delta) was expected to prevent this from occurring. Recently, a few mid-term clinical studies on the fourth-generation ceramic have been published^17–19^. Hamilton et al.^17^ reported 0.9% (3 hips) of ceramic fractures among 345 THAs at a mean 5.3-year follow-up. Lim et al.^19^ reported 0.3% (2 hips) of ceramic liner fractures without malseating among 749 THAs at a mean 6.5-year follow-up. Overall, the rate of the fourth-generation ceramic factures appears to be low in comparison to the reported rates of the third generation ceramic fractures ranging from < 1 to 4.4%^20–22^. In this study, ceramic fracture was found to be a major cause of failure in the third-generation CoC THAs, whereas it was not in the fourth-generation CoC THAs [31/92(33.7%) *vs*. 3/54(5.6%)].

Squeaking is another concern of CoC bearings. Previous studies have shown that the occurrence rate of squeaking in the third-generation ceramic bearing varies from 0.7% to 20.9%^23^. Stanat and Capozzi^24^ conducted a meta-analysis on the third-generation CoC THAs and yielded a squeaking incidence of 2.4%. Zhao et al.^25^ recently analyzed 3689 THAs in which the fourth-generation ceramic bearing was applied. Their reports found that squeaking occurred at a rate of 3% in the fourth-generation CoC bearing, suggesting a lack of superiority of the fourth-generation ceramic in terms of squeaking. In our study, the overall incidence of squeaking was 4.1% (6/146), all of which occurred with the third-generation CoC bearing.

The femoral head size plays a role in ceramic fracture and also in squeaking. A 28 mm head size has a higher fracture rate, compared to larger sized heads^26^. A short neck length similarly has a higher risk of fracture compared to longer neck^27^. These phenomenon were believed to result from a reduced distance between the corner of the bore and outer surface of the head which can predispose to crack propagation. In our series, ceramic fracture was a major reason for 28 mm-head THA revisions. According to Tai et al.^28^, in their series, 7.3% (15 of 206 hips) of the hips were recorded as squeaking. Levy et al.^29^ suggested that a higher incidence of squeaking in larger heads is primarily due to the increase in the total work done at the articular interface correlated to the applied friction force.

The general taper angle of acetabular cups for ceramic liner is 18°, however, for acetabular shells with multiple options (metal, polyethylene, ceramic), the taper angle is lower. A recent study showed that the risk of malseating a ceramic liner is significantly higher for metal shells with lower taper angle compared to shells with a 18° taper^30^. This is a concern on fracture risk of ceramic liners.

An important feature of retrieved Biolox delta is the metal transfer on femoral head. It generally implies a meaningful alteration of the bearing surface^31^. The mechanisms of metal transfer were known as femoral head dislocation, closed reduction procedures, impingement, or third body entrapment in the articulating zone. In particular, it has been hypothesized that metal transfer on the femoral head is associated with joint instability and subluxation/dislocation^32^.

Nevertheless, some advantages of ceramics led to continuous increase in use of CoC bearings in THA. CoC bearings guarantees the complete avoidance of metal debris and also, reduce the risk of wear-induced osteolysis^33^. The CoC bearings showed very low friction and very low wear rates. The wear is not directly dependent on the head diameter which allows surgeons to select a larger diameter head with fewer concerns^34^.

In our series, younger age, lower ASA grade, and preoperative diagnosis of osteonecrosis were associated with CoC revisions from bearing-related problems. These variables are commonly connected to high daily activity. Younger age and higher activity may increase the risk of impingement and subsequent mechanical failure in THAs^35^, though there is not sufficient evidence supporting the relationship between high daily activity and ceramic fractures. The use of a 28 mm short-neck femoral head and component malposition is known to increase the risk of ceramic fracture^36^.

The most common reasons for CoC THA revisions were ceramic fracture and aseptic loosening of the implanted prosthesis. The reasons for CoC revisions differed according to the generation of the ceramic bearing. In THAs with the third-generation CoC bearing, 41.3% (38 out of 92) were revised due to bearing-related problems. In contrast, only 7.4% (4 out of 54) of the fourth-generation CoC THAs were revised due to bearing-related problems. Younger age, lower ASA grade, and preoperative diagnosis of osteonecrosis were factors related to the need for CoC revision due to bearing-related problems. When using the fourth-generation ceramic with proper design and head size of 32 or over, less revision is expected on the long-term.
